# The Physician’s Visiting List for 1885

**Published:** 1885-01

**Authors:** 


### The Physician's Visiting List for 1885.

PUBLISHED BY P. BLAKISTON, SON & CO.

This is the thirty-fourth year of its publication. This alone is good evidence of its value and popularity. In its line, it has not a superior, if an equal, anywhere. It contains, in small compas and in a condensed form, much that is of value and importance to every general physician. To the specialist it also is valuable.

It gives a list of poisons and antidotes and a dose table, either of which is worth far more than the price of the book.

A list of new remedies is given that is of interest and importance.

The work is one that should be in the hands of every one engaged in any department of medical practice. It can be had of the publishers or of booksellers generally. Price, $1.00.

An amalgam for filling teeth has been patented by Mr. Walter C. Davis, of St. Petersburg Place, Bayswater, Middlesex County, England. Amalgam filings are coated, by a special process, with a varnish of gum and gold dust, so that each individual grain or particle of the amalgam is protected from the action of the atmosphere or the acid secretions of the mouth.

